# Crk and CrkL adaptor proteins: networks for physiological and pathological signaling

**DOI:** 10.1186/1478-811X-7-13

**Published:** 2009-05-10

**Authors:** Raymond B Birge, Charalampos Kalodimos, Fuyuhiko Inagaki, Shinya Tanaka

**Affiliations:** 1 Department of Biochemistry & Molecular Biology, UMDNJ-New Jersey Medical School, 185 South Orange Ave, Newark, NJ 07103, USA; 2 Department of Chemistry, Chemical Biology and Biomedical Engineering, Rutgers University, 599 Taylor Road, Piscataway, NJ 08854, USA; 3 Department of Structural Biology, Graduate School of Pharmaceutical Sciences, Hokkaido University, N-21, W-11, kita-ku, Sapporo, 001-0021, Japan; 4 Department of Pathology, Division of Medicine, Hokkaido University School of Medicine, North 15, Kita-ku, Sapporo, 060-8638, Japan

## Abstract

The Crk adaptor proteins (Crk and CrkL) constitute an integral part of a network of essential signal transduction pathways in humans and other organisms that act as major convergence points in tyrosine kinase signaling. Crk proteins integrate signals from a wide variety of sources, including growth factors, extracellular matrix molecules, bacterial pathogens, and apoptotic cells. Mounting evidence indicates that dysregulation of Crk proteins is associated with human diseases, including cancer and susceptibility to pathogen infections. Recent structural work has identified new and unusual insights into the regulation of Crk proteins, providing a rationale for how Crk can sense diverse signals and produce a myriad of biological responses.

## Introduction

**Figure 1:**
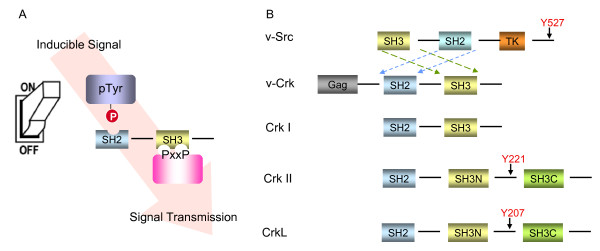
**(A). Coupling of signals through modular SH2 and SH3 domains**. Signals are initiated via extracellular factors that induce intracellular tyrosine phosphorylation (indicated by the light switch) which are subsequently relayed to downstream targets through SH3 binding partners (indicated by arrowhead). (B). Structure of the Crk family of proteins. The domains are boxed: SH2, Src homology 2; SH3, Src homology 3; Gag, viral group specific antigen; Y221 or Y207, negative regulatory phosphorylation site. The structure of Src is shown at the top of the figure to indicate its spatial arrangements compared to Crk. TK, tyrosine kinase domain.

Crk has a story book history, first emerging in the late 1980s as a novel retroviral gene product, v-Crk or Gag-Crk, and later serving as a major impetus for unraveling how modular protein domains assemble into organized protein-protein networks during signal transduction. The study of its mechanism of action has been full of unexpected and interesting findings, beginning first with a paradox as to how an oncogene product without intrinsic tyrosine kinase activity strongly and selectively increases cellular tyrosine phosphorylation levels. v-Crk and its cellular homologs, Crk II, Crk I, and the paralog CrkL, comprise the prototype of a novel class of regulatory proteins, called adaptors, composed of modular Src Homology 2 (SH2) and Src Homology 3 (SH3) domains separated by flexible linker sequences that act as building blocks to assemble multiprotein complexes. SH2 domains are structurally conserved protein domains of ~100 amino acids contained within the Src oncogene and other signaling proteins that bind tyrosine phosphorylated proteins in the context of short peptide sequences and localize SH2 domains to tyrosine phosphorylated proteins. SH3 domains are structurally conserved domains of ~60 amino acids that bind a consensus sequence of X_1_-**P**_2_-p_3_-x_4_-**P**_5 _where 1 and 4 are aliphatic amino acids, 2 and 5 are always proline, and together this sequence binds to the hydrophobic pocket of the SH3 domain. There are over 110 SH2 domains and 300 SH3 domains in the human genome, making this general signaling strategy widely utilized in metazoan cells to transmit intracellular signals. As the name adaptor implies, these molecules physically bridge tyrosine phosphorylated proteins to various intracellular signaling pathways (Figure 1A). An impressive body of work over the past two decades has demonstrated that the signal transduction functions of v-Crk, c-Crk, and CrkL are attributed to the formation of coordinately regulated protein complexes that bind to the SH2 and the more N-terminal SH3 domain (SH3N) (Figure 1B). The Crk SH2 domain binds short tyrosine phosphoryated proteins in the context of pTyr-Asp-x-Pro, and the SH3N domain binds to proteins with signature proline-rich sequences in the context of Pro-x-x-Pro-x-Lys/Arg (where x is any amino acid). To function as an adaptor protein, both the SH2 and SH3N domains need to be operational in time and space, acting as molecular adhesives to draw disparate information together to spatially and temporally regulate signal transduction pathways.

### v-Crk, c-Crk, and CrkL family members

The Crk gene product was identified approximately 20 years ago in the form of a transforming gene (Gag-Crk) encoded in the genome of a defective avian sarcoma retrovirus called CT10 (chicken tumor Virus 10) [1,2]. A year later, Vogt and colleagues characterized an independent avian retrovirus (called ASV-1) isolated from a spontaneous tumor in an adult chicken with an oncogene product virtually identical to Gag-Crk [3]. The most outstanding feature of CT10- or ASV-1 transformed chicken embryo fibroblasts (CEFs) results from a selective increase in tyrosine phosphorylation of cellular proteins of pp70 and pp130, initially suggesting CT10 virus encoded a tyrosine kinase oncogene [4,5]. However, there were clear differences in the molecular characteristics of the CT10 virus compared to viruses that encoded tyrosine kinases, including the lack of induction of a classic "refractive morphology" in CEFs transformed by Rous sarcoma virus (RSV). Moreover, Northern blotting against a battery of 19 DNA fragments from previously characterized RNA tumor viruses, including many of the known tyrosine kinase oncogenes, did not hybridize with CT10 viral fragments, suggesting that the oncogene in CT10 was different from all previously identified tyrosine kinase oncogenes [2]. Subsequently, a cDNA encoding the CT10 transforming gene was isolated and shown to encode a novel gene product containing viral gag sequences fused to sequences similar to those previously identified in the N-terminal regulatory region of the Src family kinases. Because v-Crk lacked intrinsic tyrosine kinase activity, Mayer and colleagues named the new gene with an acronym for its proposed function as a transactivator of a cellular tyrosine kinase, called Crk or CT10 regulator of a tyrosine kinase.

Following the cloning of *v-crk *or *gag-crk*, Reichman, et al. reported the isolation of the cellular homolog of Crk by screening a chicken brain library using a portion of the v-*crk *probe devoid of the retrovirus-derived gag sequences [6]. Molecular cloning of chicken cellular *crk *(c-*crk*) revealed a molecule with similar structural organization to v-Crk, but containing a ~50 amino acid proline-rich linker and an additional C-terminal SH3 domain (SH3C) (Figure 1B). Matsuda and colleagues characterized two species of Crk in mammalian cells, Crk II and Crk I, derived from alternative splicing from a single gene locus [7]. Structurally, Crk I is more analogous to v-Crk and, biologically, significantly more transforming than Crk II when ectopically expressed in NIH 3T3 cells. The oncogenic activation of Crk by C-terminal truncation in the retroviral genome is reminiscent of the mode of oncogenic activation of tyrosine kinases, including the activation of Src in the RSV genome [8,9]. Indeed, it is now clear that the differences in transforming activities of Crk II and Crk I rely, at least in part, on a specific post-translational modification of Crk that involves phosphorylation on Y222 in chicken (Y221 in mouse and human) [10]. Both Crk II and CrkL are negatively regulated by autoinhibitory phosphorylation events that ultimately block the ability of Crk to function as an adaptor protein. This is analogous to the C-terminal tyrosine phoshorylation of Src family kinases by C-terminal Src Kinase (CSK) [11,12], which allows Src to adopt an intramolecular pTyr-SH2 "closed" structure involving the Src SH2 and a C-terminal phosphorylation motif [13]. Phosphorylation of Crk on Tyr 221 (or CrkL on Tyr 207) causes intramolecular binding of the linker region to the SH2 domain, sequestering the SH2 and SH3N and preventing them from binding target proteins [14,15]. Subsequent studies have shown that the negative regulation of Crk II occurs directly via the activity of the proto-oncogene products Abl and Arg tyrosine kinases, the former originally characterized from the Abelson murine leukemia virus (A-MuLV) [16]. Abl and Arg appear to be the predominant tyrosine kinases that negatively regulate Crk II (and CrkL) in cells, as CrkY221 phosphorylation is virtually undetectable in Abl/Arg (-/-) double knockout cells. Current theory holds that v-Crk and Crk I are more transforming than Crk II, mainly because their C-termini end prior to the regulatory Y221 site, although there are mutations in the v-*crk *gene that also may contribute to oncogenicity [16]. Hence, v-Crk has no clear mechanism to downregulate signals, essentially being locked as a constitutively active adaptor in signal transduction.

### Crk effector pathways: Molecular multi-tasking and signal plasticity

The Crk and CrkL proteins have central roles in an astonishingly vast number of biological processes, ranging from cell proliferation, cell adhesion and migration, phagocytic and endocytic pathways for apoptotic cells and parasitic organisms respectively, apoptosis, and regulation of gene expression [17]. Until the introduction of siRNA technology and knockouts in genetically amenable systems, the majority of studies on Crk relied on identification of binding proteins to specific domains or by overexpression of wildtype or dominant negative Crk proteins in reconstituted systems. Because the SH2 and SH3 domains are modular and retain their binding specificity and affinity when expressed as isolated fragments [4,18], GST (glutathione S transferase)-pull-down assays, Far-Western blotting, and yeast two-hybrid approaches permitted the initial characterization of Crk binding partners and inference of the participating signal transduction pathways.

Over the past two decades, over 40 cellular proteins have been identified that bind to the SH2 and SH3N domains, and for this reason, it is clearly daunting to assign one specific cellular function to Crk [17]. Indeed, a PubMed search combining "Crk" and "signaling" returns over 700 articles, and survey of this literature clearly indicates that the adaptor function of Crk is used repetitively and often as a signal transduction strategy in the context of both physiological and pathophysiological situations (Table 1; and references [2,5,19-73]). Despite such complexity in Crk transduction outcomes, some general rules apply regardless of the specific proteins involved. First, the Crk SH2 domain (the input pathway) binds phosphotyrosine-containing proteins in an inducible on-and-off switch mechanism that involves regulated tyrosine phosphorylation and dephosphorylation. Second, the interactions between the Crk SH3N domain (the output pathway) occur constitutively, and these complexes are generally regulated without post-translational modifications, although there are a few exceptions whereby tyrosine phoshorylation has been shown to impinge on SH3-proline-rich assemblages [74]. Therefore, while assigning functions to Crk, it is important to keep in mind that input signals depend on the availability of extracellular hormones and growth factors, the prior history of the cell, including whether the cell has been stressed or re-stimulated, the state of proliferation or differentiation of the cell, and other temporal and spatial epigenetic factors that reflect the physiological and metabolic status of the cell. As the input signals reflect changes in the cellular environment, Crk signals are subject to dynamic and plastic regulation, allowing for rapid and dynamic fluxes through signal transduction pathways that permit pleiotropic changes in the signaling capabilities of cells. Despite a detailed understanding of the types of interactions that exists between Crk and its substrates, there is still a paucity of information as to how these signals are integrated in real time and space.

**Table 1:** Examples of cellular and extracellular stimuli that result in Crk-mediated signal transduction:

**Signals**	**Tyrosine phosphorylation connection**	**Biological outcome**	**References**
**Cellular stimulation**			

v-Crk	p130Cas, pp110, paxillin	Cellular transformation	Mayer et al (1988) Matsuda et al (1990)

v-Src	p130Cas, paxillin, others	Cellular transformation	Sakai et al (1994)

Bcr-Abl	p130Cas, PI3-kinase	Cellular transformation, CML	Sattler et al (1996)

**Growth factor/Tyrosine kinase**			

NGF/TrkA	TrkA, p130Cas	Differentiation, neurite outgrowth	Hempstead et al (1994) Ribon et al (1996)

EGF/Her proteins	Cbl, p130Cas, BRK	Proliferation, receptor internalization	Fukazawa et al (1996) Ojaniemi et al (1997)

Insulin/IGF-1/IR/IGF-R	p130Cas, IRS-1, 4BS	Mitogenic Signaling	Beitner-Johnson et al (1996), Jin et al (2000)

PDGF/PDGFRα	PDGF-Rα, p130Cas	Mitogenic Signaling	Anderson et al (1990), Yokote et al (1998)

VEGF/VEGFR	p130Cas	Motility in endothelial cells, Angiogenesis	Salameh et al (2005)

HGF/Met	Gab1, PI3-kinase	Epithelial morphogenesis	Garcia-Guzman et al (1999)

Ephrin/EphB2	p130Cas, Cbl	Membrane ruffling, tumor growth	Noren et al (2006)

**Cytokines/Receptors**			

IL-8/IL-8R	p130Cas	Cytoskeletal changes	Schraw et al (1995)

IL-3/IL-3R; Epo-EpoR	Cbl	Proliferation	Barber at al (1997)

Ang-2/Tie2	p130Cas	Vascular smooth muscle cell migration	Takahashi et al (1998)

Calcitonin-Calcitonin-R	p105CasL	Cytoskeletal changes	Zhang et al (1999)

IL-2/IL2R	STAT-5	Nuclear translocation/Transcription	Oda et al (2000)

TGF-β/TGF-β1R	p130Cas	E-Cadherin cell to cell contact	Kim et al (2002)

SLDF-1	p130Cas./JAK2	Cellular migration	Zhang et al (2001)

Reelin/ApoER2	Dab1	Neuronal positioning	Chen et al (2004)

Crosslinking TCR activation	p130Cas, Cbl, ZAP70	Proliferation/Clonal expansion	Sawasdiksol et al (1995), Buday et al (1996)

LFA/BCR activation	p130Cas, Cbl	B cell activation/differentiation	Petruzzelli et al (1996)

LPA/Bombesin/RhoA	p130Cas, paxillin	Stress fiber formation/focal adhesions	Flinn et al (1996)

GABA/m3 muscarinic-R	FAK, p130Cas	Exocytosis (pancreatic cells)	Rasado et al (2000)

CCL20/CCR6	p130Cas	Epithelial cell adhesion/migration	Yang et al (2005)

**Cell adhesion/Integrins/Mechanical force**			

FN/Integrin β1	p130Cas/FAK	Cell adhesion and migration	Nojima et al (1996) Armulik et al (2000)

uPA/uPAR	p130Cas	Motility in tumor cells	Smith et al (2008)

MCSP/α4β1 integrin	p130Cas	Tumor invasion	Eisenmann et al (1999)

Shear/Mechanical tension	p130Cas	Src activation/Focal adhesion remodeling	Okuda et al (1999), Sawada et al (2006)

Col/Col-R	Paxillin	Cell Dispersion	Petit et al (2000)

Osmotic Stress/Shock	Gab1, p130Cas	Stress responses	Gaul et al (2002)

ECM proteins/Integrins	p130Cas	Rac1-mediated motility	Cho et al (2000)

Laminin 10/11	p130Cas	Cellular migration	Gu et al (2001)

TIMP-2	Paxillin	HVEC motility and invasion	Oh et al (2006)

Chondroitin-Sul/Neuropilin	p130Cas	Axon guidance	Liu et al (2007)

HLA	ERM proteins	Cell migration	Tsuda et al (2004)

**Particles**			

Apoptotic cells	p130Cas	Phagocytosis	Reddien et al (2000), Albert et al (2000)

Molecular patterns/CD36	p130Cas	Phagocytosis	Stuart et al (2007)

Adenovirus/αvβ5 integrin	p130Cas	Virus endocytosis/trafficking	Li et al (2000)

Yersinia	p130Cas	Pathogen uptake	Weidow et al (2000)

Shigella	p130Cas/Abl	Bacterial uptake	Burton et al (2003)

Listeria/InIB receptor	Gab1	Bacterial Entry	Sun et al (2005)

Helicobactor	CagA	Entry into Gastric cells	Suzuki et al (2005)

**Other**			

Fluoroaluminate	p130Cas	Spreading of pre-osteoblstic cells	Freitas et al (2002)

Shingosine-1-phosphate	p130Cas	Gi-mediated motility	Ohmori et al (2001), Endo et al (2002)

MFG-E8	p130Cas	Phagocytosis	Akakura et al (2004), Hamayama et al (2002)

Estrogen/Tamoxafin	Gab1, p130Cas	Non-genomic actions of E2	Cabodi et al (2004), Cowell et al (2006)

The data are arranged in potential classes of initiating signals, as well as major tyrosine phosphorylated scaffolds that assemble with Crk or CrkL. For specific examples, please see references [2,5,19-73] in the bibliography.

Another important aspect of Crk signaling that requires definition is to assess which signaling pathways depend exclusively on Crk or CrkL versus those signaling pathways whereby Crk and CrkL can compensate for each other. Despite considerable homology between Crk II and CrkL in their SH2 and SH3 domains, development of knockout mouse models clearly show these gene products have distinct, non-overlapping roles, during embryonic development as both Crk knockout and CrkL knockout mice die perinatally with different developmental defects. Moreover, in Crk II (-/-) fibroblasts, CrkL proteins were not overexpressed in a compensatory manner, and vice versa of CrkII expression in CrkL (-/-) cells, again suggesting little molecular crosstalk at the level of expression in embryonic cells [75]. Interestingly, mice homozygous for a null mutation of CrkL show a phenotype characteristic of DiGeorge/velocardiofacial syndrome, in which neural crest derived cells fail to differentiate and migrate leading to defects in cranial and cardiac development [76-78]. Crk null mice (which lack both Crk II and Crk I) die perinatally due to defects in cardiac and skeletal development [79]. Imaizuma and colleagues generated another mouse using an exon trap method that lacks Crk II but still expresses Crk I. Although Crk I expressing mice show no obvious developmental abnormalities, it is not known whether these mice develop age-dependent malignancies, since they die within a few days after birth by unknown mechanisms [80].

However, in contrast to the aforementioned observations showing independent roles for CrkL and Crk II during development, a number of recent studies suggest that Crk II and CrkL are co-expressed in somatic cells and can compensate to each in cellular signaling. An example of such redundancy comes from several independent studies dissecting the Reelin signaling pathway in cortical pyramidal cells of the hippocampus. Reelin, a secreted glycoprotein that binds to receptors on hippocampal neurons such as ApoER2, VLDLR, and α3β1 integrin, controls cell positioning during adult neurogenesis via Src-dependent tyrosine phosphorylation of the scaffold protein Dab1 [81]. Upon receptor activation, Dab1 becomes tyrosine phosphorylated at multiple sites, and binds a variety of SH2 domain-containing proteins that include both Crk II and CrkL [82,83]. Studies using shRNA or cre-lox deletion of Crk and CrkL showed that knockdown of both proteins are required for the defects in neuronal positioning, and both proteins appear to converge on the activation of Rap1 through C3G (see below) [84-86]. How universal Crk and CrkL compensation is in signal transmission still remains an active area of investigation.

### Role of p130Cas and molecular scaffolds in signal integration and Crk function

Much of the early emphasis on Crk biology focused on the mechanisms of increased cellular tyrosine phosphorylation and models for v-Crk transformation. Although it is still reasonable to consider that v-Crk binds to and "hijacks" a cellular tyrosine kinase, no model in which a specific tyrosine kinase is activated has provided a unifying molecular mechanism for v-Crk and Crk I transformation. However, a number of studies indicate that expression of v-Crk in Src (-/-) or Fyn (-/-) fibroblasts significantly reduces Crk-inducible tyrosine phosphorylation [87], and such studies imply that v-Crk transformation, at least in part, requires initial activation of a Src family kinase member (SFK). Subsequently, Akagi and colleagues demonstrated that focal adhesion kinase (FAK) Y397 phosphorylation (mediated by SFKs), and the recruitment of phosphatidylinositol 3'-OH kinase (PI3K) to FAK was also required for v-Crk-dependent transformation of CEFs [88,89]. This observation is consistent with recent studies employing Src/Fyn/Yes (SFY)-deficient MEFs, whereby Src was shown to be required for FAK tyrosine phosphorylation and Crk-mediated motility [90].

With respect to the substrates of tyrosine phosphorylation and Crk transformation, a great deal of attention has been placed on the p130Cas/HEF1/Efs proteins, a family of non-enzymatic docking proteins that contain multiple interacting domains and serve as important anchoring points for protein-protein interactions [46,91]. p130Cas (Crk associated substrate), the most ubiquitously expressed member, was in fact discovered as a result of its elevated phosphotyrosine levels observed in CT10-tranformed cells [5,19,92]. The importance of p130Cas in Crk and Src transformation has been illustrated not only because p130Cas stably and persistently binds SFKs, but also from studies that tyrosine kinase and Ras oncogenes fail to transform p130Cas (-/-) fibroblasts [93,94]. Recent studies also indicate that p130cas is necessary and sufficient to transform cells, as a transgenic MMTV-p130Cas mouse that overexpresses p130Cas in the mammary gland shows extensive mammary epithelial hyperplasia during development and pregnancy with an association of elevated Src activity [95]. Moreover, a double transgenic line expressing MMTV-p130Cas and MMTV-HER2-neu developed multifocal mammary tumors at an accelerated level [95]. Overexpression of p130Cas also renders breast cancer cells more resistant to cytotoxic chemotherapies, such as anti-estrogen agents or adriamycin, indicating that p130Cas activates survival and metastatic pathways when tyrosine phosphorylated [96,97]. Indeed, these studies are consistent with the fact that highly metastatic breast cancer epithelial cells up-regulate β1 integrin and p130Cas, and show stress fiber formation that correlates with EMT and motility from the site of tumor origin [98]. Although to our knowledge v-Crk transformation has not been reported in p130Cas (-/-) cells, expression of a p130Cas substrate trap (which essentially contains just the Crk substrate region) effectively abrogates v-Crk transformation [99]. Since many tumor cells express high amounts of tyrosine phosphorylated p130Cas, these and other studies implicate p130cas as a central component for Crk transformation.

Mice with homozygous null mutations of p130Cas exhibit marked growth retardation with poorly developed heart and vasculature and die *in utero *from congestive heart failure, edema, and hemorrhaging due to breakdown in the regulation of vascular permeability [94]. Isolated and immortalized p130Cas (-/-) embryonic fibroblasts display short disorganized actin filaments, show defects in actin bundling, and these cells have small and poorly organized focal adhesions. However, a functional role for p130Cas in non-motile cell types, including neurons, is likely equally important and recent studies in Drosophila showed that targeted loss of p130Cas in CNS neurons induced defects in neurite guidance and target fasciculation [100]. In fibroblasts, v-Crk localizes primarily at focal adhesions, and for this reason, the association of Crk with events associated with p130Cas and actin cytoskeleton assemblages has gained considerable acceptance in the Crk transformation field [101,102]. Specific targeting of Crk to focal adhesions by fusing a FAT sequence to Crk induces p130Cas and FAK phosphorylation and potentiates cell migration [102].

**Figure 2:**
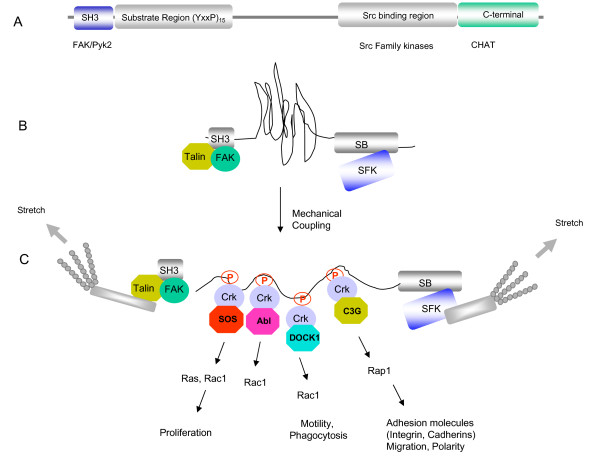
**Structural characteristics and interacting proteins of p130cas**. (A). p130Cas is a nonezymatic scaffolding protein that contains, (i) an N-terminal SH3 domain that binds FAK and Pyk2, 15 repeats of a YxxP motif, a serine-rich motif that binds Src kinases, and a conserved C-terminal region that binds members of the Chat family of proteins. (B). Signal transduction by the p130Cas scaffold protein. The central substrate region of p130Cas (shown in panel B as a compressed configuration) is activated by mechanical force and "extension" of the central region (C). This would activate Src, induce tyrosine phosphorylation of the repetitive YxxP motifs, and recruit Crk through its SH2 domain. Further, by recruiting different proteins via the CrkSH3N, this signaling strategy would spatially integrate divergent signals, for example, after the recruitment of various GTPase pathways such as DOCK1, SOS, and C3G.

At the molecular level, p130Cas has multiple protein-protein interaction domains including an N-terminal SH3 domain that binds FAK and Pyk2, an interior "substrate domain" characterized by 15 YxxP motifs, a C-terminal Src binding domain, followed by a highly conserved C-terminal region that binds to the Nsp family of proteins (Nsp1, And-34, and Chat) [91] (Figure 2A). Based on the unusual arrangement of repetitive YxxP motifs, there has been continued interest and conjecture as to the reasons why this motif has been duplicated 15 times, since when phosphorylated, they comprise a consensus binding site for the SH2 domain of Crk. Interesting studies by Miller and colleagues showed that the p130Cas substrate region was processively phosphorylated when Src bound to the polyproline-region of p130Cas, initially suggesting that the order of addition of phosphates may organize a pattern to prioritize a signaling mechanism [103]. However, their more recent studies suggests that p130Cas phosphorylation is stochastic, meaning that no single "lynchpin" permits phosphorylation at additional sites and that the phosphorylation does not appear to follow an obligatory sequence [104]. This would suggest that the juxtaposed repetitiveness of the YxxP motifs in p130Cas would mainly function in signal amplification to maximize the Crk output pathways, but also to diversify signaling, as functional cooperation in the Crk assemblages would also increase the repertoire of output signals, for example during integrin-mediated spreading and migration on extracellular matrix (ECM) molecules [105] (Figure 2C). Assuming that several of the tyrosines in the YxxP motifs are concomitantly phosphorylated at any given time, this might indicate that separate molecular complexes of p130Cas/Crk/DOCK180, p130Cas/Crk/C3G, p130Cas/Crk/Abl, p130Cas/Crk/JNK, and p130Cas/Crk/PI3K could exist simultaneously, but this remains to be seen experimentally (Figure 2C). Clearly, the development of site-specific, phospho-specific antibodies aimed to quantify and map the timing and sequence of p130Cas phosphorylation events could be used to determine whether there are more subtle patterns in the motifs that are phosphorylated, or perhaps more importantly, whether the half lives of individual phosphorylation events vary during signal downregulation. Such multiplicity in p130Cas phosphorylation could achieve extraordinary diversification in signaling by acting as a molecular scaffold to transmit localized subcellular signals.

The biological role of p130Cas phosphorylation is also intriguing from the vantage point that a vast variety of extracellular matrix molecules, growth factors, hormones, and metabolic intermediates that induce tyrosine phosphorylation of p130Cas and subsequent coupling to Crk (Table 1). In addition, recent studies have demonstrated that p130Cas phosphorylation is sensitive to physical transduction by mechanical force [51] and this may provide a common element for convergence in signal transduction (Figure 2B). These most interesting studies showed that when cells are cultured on a stretchable substrate consisting of collagen-coated flexible silicone to uniformly and biaxially stretch cells by 10% or more, p130Cas was inducibly tyrosine phosphorylated within 1 minute of the stretch. Although these experiments were designed to mimic integrin-mediated events, it is important to note that tyrosine phosphorylation of p130Cas occurred in the absence of specific integrin ligation and outside to inside integrin signaling. Moreover, using a clever FRET-based assay to monitor stretching of the p130Cas substrate domain (SD) in vitro, these investigators showed extension-dependent phosphorylation of the SD by both Src and Abl tyrosine kinases, but not CSK or ZAP-70. These data suggest that mechanical stretching of the substrate region, presumably by actin-tethering proteins brought together by binding to FAK and/or Src, is sufficient to cause p130Cas phopshorylation and subsequent coupling to downstream pathways (Figure 2C). This data also suggests that physiological events that induce stretching, i.e. adhesion, motility and engulfment of large particles, as well as pathophysiological events such as hypertrophy, are probably sufficient to induce p130Cas tyrosine phosphorylation, independent of the particularities that initiate the signal.

Once extended, the central region of p130Cas is a substrate for activated tyrosine kinases, and p130Cas itself has binding sites for FAK [106] and Pyk2 [107] (via the SH3 domain) and SFK members through the C-terminal Src binding region (SBR) [21] (Figure 2A). *In vivo*, both of these kinases have been proposed to contribute to p130Cas phosphorylation, possibly to permit different mechanisms and different input signals to converge on assembling Crk linkages [108]. To gain a better perspective on the specific role of Src versus FAK in mediating p130Cas phosphorylation, and determine whether phosphorylation is sufficient to activate downstream signaling, Sharma and Mayer [109] developed an approach called functional interaction trapping (FIT). Using this strategy, two signaling molecules are brought together by engineering fusion proteins with artificial binding surfaces composed of two leucine zipper coiled-coiled domains of ZipA and ZipB. When FIT was employed to bring together full-length p130Cas or the substrate region of p130Cas and Src, p130Cas was heavily phosphorylated on tyrosine and phosphorylated 130cas was sufficient to activate Rac1 and localize to focal adhesions [109]. In apparent contrast, when FAK was fused with ZipB and expressed with p130Cas, p130Cas was weakly phosphorylated. This result appears to resolve a long-standing question as to whether FAK acts principally as a scaffold, since the Tyr397 site in FAK can recruit Src family kinase through its SH2 domain. However, functional studies show that FAK binding is important for p130Cas tyrosine phosphorylation in vivo, as fibroblasts derived from mice expressing a truncated p130Cas that lacks exon 2, and hence the N-terminal SH3 domain that binds FAK, fail to induce p130Cas phosphorylation when fibroblasts were induced to spread on FN [110].

Analogous to the theme described above for p130Cas, other scaffolding molecules that are acted upon by extrinsic signals ultimately interact with Crk, thereby launching alternative versions of a common signaling paradigm. Numerous stimuli, ranging from growth factors and cytokines to ECM, apoptotic cells, microbial products, immune complexes, and endogenous metabolic products activate trans-membrane receptors that inducibly phosphorylate proteins that, in turn, engage the Crk SH2 domain (Table 1). These proteins include Dab1 [39], IRS-1 [25], Paxillin [111], Gab1 [112], and CasL/Efs [91], and analogous to p130Cas, these proteins contain tandem YxxP motifs. As pointed out by Feller, et al., examination of the databases shows that the occurrence of three or more YxxP motifs in 100 kDa intracellular proteins is rare [17], raising the question of how such tandem elements are designed for functional crosstalk between Crk adaptor, or other adaptor protein pathways. As alluded to above, utilization of different scaffolds could allow for localized signaling, by virtue of being targeted by specific kinases, or could control the selectivity of Crk signaling. A good illustration of this comes from a study by Lamorte and colleagues, showing that a switch from a p130Cas/Crk to a Gab1/Crk complex in Met oncogene transformed cells correlates with a change from a motile phenotype to a proliferation phenotype [113]. In a few cases, Crk can interact directly with tyrosine kinase receptors. For example, direct recruitment of Crk to the PDGFRα [28] or the VEGFR-3 [29] can regulate immediate post-receptor signals from a subset of growth factors at the plasma membrane.

Once the Crk proteins are engaged by tyrosine phopshorylation via the SH2 domain in a particular time and place, they can transmit signals downstream through constitutive binding to the Crk SH3 domain. Although many Crk SH3N binding proteins have been identified and have been the subject of an excellent review [17], some of the better characterized p130Cas signaling pathways are described below.

#### Crk and C3G

C3G (Crk SH3-domain-binding guanine-nucleotide releasing factor) was isolated in a screen for Crk SH3N-binding partners and was the first protein identified that bound to the SH3 domain of Crk [114,115]. In addition to several proline-rich sequences that bind Crk, C3G has a guanine nucleotide releasing activity that removes GDP from the small GTPases Rap1 (Krev-1), Rap-2, and R-Ras, allowing their spontaneous reloading with GTP [116]. Rap-GTPases (like other Ras-GTPases) are thought to be inactive when bound to guanosine diphosphate (GDP), and activated when bound to guanosine triphosphate (GTP). As their name implies, GTPases possess intrinsic enzymatic activity that hydrolyses GTP to GDP and phosphate. Thus, upon binding to GTP, the duration of Rap-GTPase activity depends on the rate of hydrolysis. C3G (and other guanine nucleotide exchange factors) act by binding Rap-GTPases and catalyzing the release of their bound GDP nucleotide. Once released from C3G, the Ras-GTPase quickly binds fresh guanine nucleotide from the cytosol because cytosolic GTP is approximately ten times more abundant than cytosolic GDP.

Although Rap1 have strong homology to classic Ras proteins and were initially identified as native antagonists to Ras that bound to and inhibited Raf-1, Rap1 signaling is clearly complex and multifactorial, and targets of Rap-1 have been implicated in cell proliferation, cytoskeletal reorganization during cell adhesion to ECM, and for cell-to-cell contact [116]. Although expression of "activated" Rap1V12 in fibroblasts does not induce classic cell transformation analogous to RasV12, Rap1 has been implicated in increased cell proliferation and transformation in EGF-stimulated Swiss 3T3 fibroblasts that express B-Raf, and these cells can form tumors in nude mice [117,118]. In addition, studies in both mice and humans have demonstrated an important role for the RapGAP, Spa-1, in cancer development. For example, Spa (-/-) targeted mice show constitutive activation of endogenous Rap1 in hematopioetic progenitor cells, and these mice exhibit a marked increase in granulocytic cells analogous to CML, with a subset showing blast crisis analogous to CML [119]. Spa-1 polymorphisms that alter Spa-1 activity have also been correlated with a likelihood for metastastic disease in humans [120]. In addition to Spa-1 deficiency, forced expression of C3G has been implicated in T-cell acute lymphoblastic leukemia [121]. C3G also has been implicated in the activation of R-Ras, which can activate the stress-related kinase JNK1 [122,123]. Although the catalytic activity of C3G is involved in transformation in rodent fibroblasts, interestingly, sequences outside the catalytic domain of C3G also have tumor suppressor activity, and recently PP2A was shown to bind to the N-terminal region of C3G [124,125]. Finally, in chronic myelogenous leukemia (CML), a novel truncated form of C3G (p87), without the inhibitory region, was found to be associated with Bcr-Abl [126].

One of the most important biological effects of Rap1 signaling is the acute regulation of cell adhesion, in part, mediated by the activation of integrins. Recruitment of C3G to the plasma membrane links p130cas to C3G, where Rap1-GTP increases the affinity of β1 integrins to ECM, the so-called inside to outside integrin signaling [127,128]. Rap1 signaling also induces cell spreading and F-actin dynamics by binding to the Rap1-interacting adaptor molecule (RIAM) [129], which may also control E-cadherin and cell to cell interactions [130]. Functionally, C3G activity is potentiated by tyrosine phosphorylation at Tyr504 [131], mediated by SFKs, and therefore it is likely that C3G activity coincides with its recruitment to p130Cas and the activation of Src that accompany cell adhesion to ECM, following stimulation of cells with certain cytokines, as well as following stretch-inducible mechanical force. Tyrosine phosphorylation of C3G is further accompanied by its relocation from the cytosol to the cortical actin cytoskeleton and the *trans*-Golgi, additionally suggesting a role in protein trafficking or secretion [132]. In neuronal cells, one of the best-understood functions of p130Cas/Crk/C3G-induced activation of Rap1 involves the activation of B-Raf in neurons. B-Raf forms a stable complex with Rap1 and converges on a signaling pathway to sustainably activate the mitogen-activated protein kinase (MAPK) pathway to control neuroblast differentiation [133].

#### Crk and Son of Sevenless (SOS)

SOS was first identified in *Drosophila melanogaster *as a functional gene product downstream of *sevenless *in the Ras/MAP kinase pathway [134]. When *sevenless *is mutated during development of the fly's ultraviolet light-sensitive compound eye, the seventh, central photoreceptor (R7) of each ommatidium fails to form. Subsequent studies indicated that SOS was homologous to the yeast exchange factor for Ras, CDC25, and then identified as a central conduit to link receptor tyrosine kinases to Ras following growth factor stimulation in metazoan cells [135]. In mammalian cells, there are two SOS homologs, SOS1 (~170 kDa) and SOS2 (~150 kDa) derived from different genetic loci. The N-terminal of SOS1 encodes a Dbl-homology (Dbl) and Pleckstrin homology (PH) duet that exchanges GTP for GDP on Ras, as well as tandem C-terminal proline-rich motifs that interact with several adaptor proteins, including Grb2 and E3b1. Although SOS can interact with both Grb2 and E3b1 (a Rac1 GEF) adaptors simultaneously, very recent studies indicate that the timing of these reactions significantly differ in vivo, the Ras activation occurring in a rapid and transient fashion, while Rac1 activation occurring in a more sustained and protracted manner [136].

Both SOS1 and SOS2 possess repetitive proline motifs that conform to consensus Crk SH3 binding motifs and direct stable association between Crk and SOS. A functional significance for this interaction is evident from co-expression studies showing that dominant negative Ras protein suppresses v-Crk-induced cell transformation in CEFs [16]. Moreover, in Crk-I transformed NIH3T3 cells, RNAi downmodulation of SOS1 significantly decreased cell transformation (soft colony formation) and tumorigenicity when Crk-I transformed cells were xenografted in nude mice (Bruce Mayer, personal communication). Finally, it is also interesting to point out that the major deficiencies observed in Crk null mice (namely cardiovascular and cranial defects) are also observed in subsets of patients with Noonan Syndrome. Noonan syndrome is a congenital syndrome characteristic of multiple abnormalities that include facial dysmorphology and cleft palate, short stature, and hypertrophic cardiomyopathic defects that include defects in the vascular smooth muscle architecture [137]. In about 15–20% of the clinical cases of Noonan syndrome, SOS1 mutations are present throughout the coding region of the gene that directly influence the GEF activity [138]. Future studies aimed at crossing SOS1 mutant with Crk (-/-) and Crk transgenic mice may shed additional light on how Crk and SOS interact with respect to tissue-specific functions.

#### Crk and DOCK180 (DOCK1)

DOCK180 (Downstream of Crk; DOCK1) was originally cloned by far-Western blotting, with Crk SH3N domain as a "bait" molecule [139,140], and represents the third GEF that directly associates with the Crk SH3 domain. However, unlike SOS and C3G exchange factors, DOCK1 does not contain the conventional DH-PH duets known to promote nucleotide exchange. Instead, DOCK1 mediates nucleotide exchange via a novel evolutionarily conserved Docker/DHR2/CZH2 domain that recruits ELMO, which functions in trans as a bipartite Rac-1-GEF [141,142]. Although a complete understanding of the structural mechanisms for Rac1 activation are still emerging, biochemical studies indicate that DOCK1 must interact with ELMO for efficient Rac1 activation. The prototypical member of the ELMO family is ELMO1 that is characterized by N-terminal Armidillo repeats, and a PH domain and proline-rich sequences towards the C-terminus.

**Figure 3:**
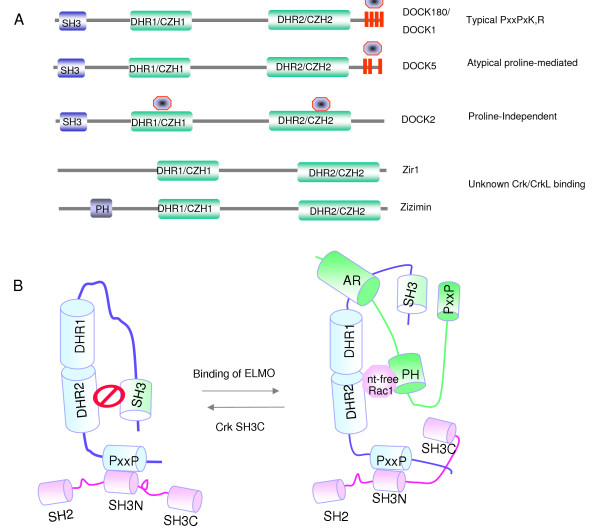
**(A). Schematic structures of DOCK proteins**. The SH3 domain, Docker Homology Region 1/CDM-Zizimin Homology1, Docker Homology Region 2/CDM Zizimin Homology 2, and Proline-rich sequences (shown in red) are indicated. The circular structure indicates the putative location of the Crk binding sites. Note that in DOCK5, Crk has been shown to bind to a non-canonical proline sequence. In DOCK2, Crk has been proposed to bind independent of proline. (B). Structure of the autoinhibited DOCK1/Crk complex. See text for details.

The bi-molecular nature of the DOCK1/ELMO Rac-GEF argues that, in contrast to conventional Rac-GEFs in which the DH and PH domains act as cis-acting elements, a significant aspect of the regulation of DOCK/ELMO mediated catalysis depends on the regulation of the association between DOCK1 and ELMO proteins, as well as other trans-acting factors (Figure 3B). In this respect, there are at least three known mechanisms by which DOCK1 and ELMO interact, one involving a PxxP-dependent SH3 domain interaction between the C-terminus of ELMO and the SH3 domain of DOCK1, the second involves atypical elements within the C-terminus of ELMO that interact with non-proline rich motifs in the N-terminus of DOCK1, and third, the ELMO PH domain interacts with nucleotide-free Rac1. Recent evidence suggests that several (if not all DOCK family proteins) have the capacity for structural inhibition mediated by an atypical SH3 domain interaction between the N-terminal SH3 domain and the DHR2 region [142]. Binding of ELMO to the SH3 domain of DOCK1 would not only relieve the inhibition of DHR2 and permit Rac1 to bind to this region, but may also permit PH mediated catalysis. Therefore, one of the important strategies to identify the relevance of DOCK180 and ELMO is to recognize the relevant upstream and downstream effectors. A clue into such a pathway was shown in recent studies that tyrosine phosphorylation of ELMO (by Src family kinase) is required for Rac-GEF function. In addition, recent studies indicate that a newly characterized phosphatidylserine receptor called BAI can directly interact with DOCK1 [143], and the RhoG GEF Trio can directly interact with ELMO [144]. Finally, binding of ELMO to DOCK1 inhibits the ubiquitylation and degradation of DOCK1 [145], suggesting that DOCK proteins may also be regulated by protein stability.

Despite the fact that DOCK1 was identified by virtue of its direct interaction with Crk, it is still not entirely clear whether Crk binding to DOCK1 directly mediates Rac1 activation at the biochemical level [60,146]. In this respect, Crk appears to have a secondary function to regulate Rac1 activation [147] mediated by the SH3C domain [148]. Mutations in the SH3C domain of Crk prevent the regulated turnover of the DOCK1/ELMO complex, suggesting that Crk also controls assembly of the ELMO/DOCK1 complex. Despite these findings, more recent functional studies employing LR73 fibroblasts showed that a direct interaction of Crk II with DOCK1 was dispensable for both engulfment of apoptotic cells as well as for the recruitment of DOCK1 since expression of mutant DOCK1 proteins that lack the proline-rich sequences were sufficient for supporting Rac1 activation [147]. This result is interesting in that it contrasts earlier genetic studies identifying Crk II (ced-2) in a linear pathway with DOCK1 (ced-5) (see below) for motility and for engulfing apoptotic corpses. Moreover, several studies have shown that dominant negative Crk proteins block migration towards certain ligands. For example, A549 lung adenocarcinoma cells and ECV304 endothelial-derived cells adhering to laminin-10/11 show more robust Rac1 activation compared to these same cells on vitronectin, and further dominant negative Crk proteins that don't interact with DOCK1 block Rac1 activation [55]. Taken together, these data imply that there may be Crk/DOCK dependent and Crk/DOCK independent interactions that depend on the initiating ligands and upstream tyrosine kinases that become activated.

Studies over the past several years have indicated that DOCK180/DOCK1 is the prototype of a superfamily of at least 11 homologous members divided into 4 subfamilies based on conservation in the DHR1/CZH1 and DHR2/CZH2 domains as well as their specificity towards Rac1 or Cdc42 [149,150]. This larger family, called CZH [CDM (CED/DOCK/Myoblast City)-Zizimin homology] can be further divided into either DOCK related (specific for Rac1) or Zizimin related (specific for Cdc42) [151] (Figure 3A). In terms of relevance to Crk and CrkL, to date none of the zizimin proteins possess classic PxxPxK, R motifs that bind Crk SH3 domains, and of the DOCK families, DOCKA (which consists of DOCK1, DOCK2, and DOCK5), and DOCKB (which consists of DOCK4 and DOCK3/MOCA), only DOCK1 has conventional Crk SH3 binding sites. However, very recent studies indicate that DOCK5 participates in Caco-2 cell motility by binding Crk L and Crk II SH3 domains via atypical proline-rich motifs in their carboxyl-termini [152]. Similar interactions between DOCK and Crk proteins outside of the classical PxxPxK motifs have been reported for DOCK2, which lacks the classic C-terminal region for binding Crk, and appears to bind CrkL via regions of the DOCKER domain [153]. Recent studies also suggest that DOCK5 cooperates with DOCK1 for the differentiation of myofibers [154], and clearly, crossing these mice with Crk II (-/-) or CrkL (-/-) mice should better define the level of functional interactions. If this is generally true, then the role of Crk signaling in regulating DOCK-C and DOCK-D family members should be revisited.

#### Crk and JNK

In addition to the ability of Crk to activate JNK through C3G and R-Ras noted above, Crk II can also interact with JNK directly through a proline-rich sequence in JNK and the SH3N domain of Crk [155]. Similar to the case for C3G and DOCK180, recruitment of a Crk/JNK complex to p130Cas may result in the localization to JNK to its relevant upstream kinases, such as MKK4 and HPK-1 [122]. A direct interaction between Crk and JNK may indeed be biologically relevant, since studies by Girardin and Yaniv have shown that a JNK mutant (K340A) that fails to bind Crk is also defective in EGFR signaling and Rac1 activation [155]. These studies also suggest significant crosstalk between JNK and Rac1, and the involvement of DOCK180 in this pathway should be investigated. These results may offer an explanation for the observations that integrins regulate progression through the G1 phase of the cell cycle in a JNK-dependent manner [156]. Following integrin engagement, JNK activation requires association of FAK with a Src kinase and p130Cas, the phosphorylation of p130Cas, and subsequently, the recruitment of Crk. FAK-JNK signaling appears to be necessary for proper progression through the G1 phase of the cell cycle. These findings suggest a role for p130Cas and Crk in both the activation of JNK and control of the cell cycle, as well as identification of a physiological stimulus for JNK signaling that is consistent with the role of Jun in both proliferation and transformation. There also appears to be significant crosstalk between these pathways. Finally, in *Drosophila melanogaster*, downstream of platelet-derived growth factor receptor (PDGFR) or vascular-endothelial growth factor receptor (VEGFR), Crk/DOCK180/ELMO/Rac interactions mediate JNK activation, leading to dorsal closure [157].

#### Crk and Abl

Abl is a non-receptor tyrosine kinase first identified as the cellular homolog of the v-Abl oncogene product of the Abelson murine leukemia virus, and has multiple roles in cell growth, transformation, cell stress, apoptosis, and remodeling of the actin cytoskeleton. Arg (Abl-related gene) is over 70% homologous to Abl, and both proteins contain four tandem proline-rich motifs located just C-terminal to the kinase domain [10,158] that mediate binding to the N-terminal Crk SH3 domain. Systematic mutagenesis studies whereby each of the four proline-rich motifs was assigned function and SH3 domain specificity showed that motifs 1, 2, and 4 (from the N-terminus to the C-terminus) predominantly bound Crk, whereas motif 3 was more promiscuous and bound SH3 domains of Nck, Ponson/CAP, and ArgB2 [159]. Moreover, in reconstitution studies in NIH3T3 cells, it was further shown that binding of Crk and Nck promoted distinct morphological phenotypes upon adhesion to ECM; Crk stimulated lamellipodia formation (via Rac1) while Nck stimulated filopodia formation (via Cdc42). This suggests that binding of Crk to the Abl proline-rich motifs is balanced to fine-tune actin-based membrane dynamics as cells adapt to their surrounding environment. This is likely functionally important for integrin function, as cell adhesion and integrin ligation induces p130Cas tyrosine phosphorylation and the translocation of Abl from the nucleus to focal adhesions, where focal adhesion-associated Abl contributes to adhesion-dependent actin reorganization and membrane ruffling [160,161]. One of the critical functions of Abl in focal adhesions is to regulate the formation and maintenance of adherens junctions via a Crk and Rac1 pathway that regulates cadherin-catenin adhesion complex [162]. Additionally, Abl has been shown to tyrosine phosphorylate SOS1, which associates with Abl most likely through Crk [163]. This might explain why Rac1 is constitutively activated in Bcr-Abl transformed cells, whereby Rac1 is implicated in increased motility of the cancer cells.

**Figure 4:**
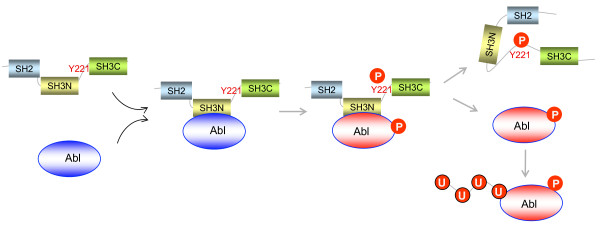
**The multiple fates of Crk and Abl interactions**. Crk binding to the proline-rich region of Abl induces Abl transactivation (indicated by circle enclosing The (P). Subsequent to Abl activation following Crk binding, Abl phosphorylates Crk on Tyr-221, causing dissociation of the Abl and Crk complex. The fate of activated Abl is not known, but some studies indicate that activated Abl is ubiquitinated and degraded by the proteosomal pathway.

In general, the interaction between Abl and Crk is suggested to be bi-directional, meaning that Crk can both transactivate Abl and Abl can transinhibit Crk II and CrkL. Transactivation of Abl by Crk II was first reported by Shishido and Hanafusa based on observations that co-expresion of Crk and Abl increased the net tyrosine phosphorylation of cellular proteins in Abl-expressing cells (Figure 4A). Subsequently, Reichman et al showed that Crk expression with Abl induced tyrosine phosphorylation of the major autophosphorylation sites in Abl (Tyr 245 and Tyr 412), suggesting that Crk binding to Abl directly enhances Abl enzymatic activity. Consistent with this idea, Crk transactivation is much more efficient when either the Y221 or the P225 motifs in Crk are substituted to prevent Crk phosphorylation and intramolecular binding to its own SH2 domain [164,165]. Although structural analysis is warranted to identify specific features of Abl transactivation, biochemical studies indicated that transactivation is mediated by the SH3 linker in Crk as well as the PNAY motif in the RT-loop of the C-terminal SH3 domain. It will also be important to decipher whether Crk mediated transactivation induces phosphorylation on specific substrates, as this would help elucidate functional significance. Biochemical and cell biological studies also suggest that Crk binding to Abl causes an increase in the steady state levels of Abl [165], possibly to prevent degradation. Abl is known to be degraded by either ubiquitin or caspase-mediated pathways. *In vitro*, addition of the Crk SH3N domain can reverse the inhibition of Abl activity by F-actin, further suggesting that Crk transactivation may occur locally in the focal adhesions [166].

Subsequent to the binding and transactivation of Abl by Crk, Crk Y221 is itself an excellent substrate for Abl, and binding of Crk to Abl reflects a potent downmodulatory signal [108]. However, while phosphorylation of Tyr221 may be the ultimate fate for the Crk/Abl interaction, few studies have attempted to understand in detail what happens between the initial binding of Crk to Abl and the ensuing Abl-mediated Crk phosphorylation. This should be an important avenue of future investigation with goals to identify transition state conformers of Abl and Crk, as well as, to attempt to generate various activation state mutants of Crk or Abl that reflect these transition states. For example, in rodent and human Crk, binding of Crk to Abl causes conformational changes that expose a PPPP motif in the Crk SH2 domain that in turn binds the SH3 domain of Abl to stabilize the Abl/Crk interaction [167,168]. Moreover, virtually nothing is known about how Crk binding to Abl influences the C-terminal region of Abl, including the ability of Abl to bind F-actin or to DNA. Clearly, one important function of Y221 phoshorylation is to prevent unregulated transactivation of complexes involving Crk and Abl. However, physiologically, it is more likely that the dynamic state of activation and deactivation of Abl by Crk represents another example of dynamic regulation to fine-tune signals related to Rac1 activation, actomyosin plasticity, and motility.

Tyrosine phosphorylation of Crk on Y221 is also not of inconsequence with respect to signal transduction pathways initiated by integrins and growth factors that led to p130Cas phosphorylation. Klemke and colleagues were the first to demonstrate that Abl inducible Crk Y221 phosphorylation acted as a molecular switch to dissociate a p130Cas/Crk/DOCK180 ternary complex, in order to block motility and invasive behavior of human pancreatic cancer cells [54] (Figure 4B). More recently, a similar negative regulatory network has been reported for a p130Cas/Crk/C3G ternary complex, whereby Abl promotes Crk Y221 phosphorylation and disrupts the interaction between Crk and C3G. This subsequently leads to decreased activation of Rap1-GTP, decreased affinity of β1 integrin to ECM, and loss of cell adhesion [169]. Abl also appears to have a negative regulatory role in HGF-stimulated hepatocytes, where Crk Y221 phosphorylation inhibits complex formation between Crk and Cbl [170]. Interestingly, in tumor cells tyrosine phosphorylation of Crk by Abl may underscore a novel anti-oncogenic pathway. For example, Noren et al have shown that EphB2 receptor tyrosine kinase acts as a tumor suppressor in breast cancer cells and that depletion of EphB2 or its ligand ephrinB2 prediposes breast cancer cells to invasion and metastasis [31]. These investigators further found that ephrinB2 induces Crk Y221 phosphorylation and decreased Rac1 activation. While this indicates that under some conditions Abl-mediated Crk phosphorylation may inhibit oncogenicity, it does raise a paradox as to whether Imatinib (Gleevec), may have side effects when used to treat CML.

Finally, a very interesting study by Peterson and Long identified an important functional role in inhibitory signaling downstream of the human killer cell Ig-like receptor (KIR) that inhibits Natural Killer (NK) cell activation and cytotoxicity [171]. Using a clever experimental system whereby NK cells either activate or inhibit target cell killing through a HLA class I receptor, these investigators determined that the killing phenotype correlated with the assemblage of a Cbl/Crk/C3G complex, whereas the inhibitory phenotype correlated with the formation of a complex with Abl and Crk, and the phosphorylation of Crk to disassemble the aforementioned complex with C3G [171]. This study defines an unusual role for Abl and Crk assemblages in the signaling of inhibitory receptors.

Recent studies also suggest that pY221Crk is not necessarily a biologically inert species. Interesting work by Vuori and colleagues showed that pY221 Crk was required for recruiting activated Rac1 to the membrane fraction, and Rac1 re-localization was prevented by expression of the Y221F Crk mutant [172]. Most interestingly, targeting of CrkY221F to the plasma membrane by fusing a CAAX box rescued the ability of Crk to localize Rac1 and promote laemellipodial formation and migration. Further support of a dynamic, rather than a static, role of Crk Y221 phosphorylation came from studies showing that Y221F Crk II expression prevents regulated turnover of Crk/Abl/p130Cas or Crk/Abl/paxillin complexes, leading to impaired adhesion and migration [173]. Taken together, these data suggest that regulated phosphorylation of CrkY221 by Abl and Arg are not a simple on-and-off mechanism, but rather a dynamic process to execute Crk-dependent signaling involving Rac1 activation and cell migration.

### Regulatory role of the C-terminal region of Crk II and domain-domain communication

Since the discovery of v-Crk and c-Crk II, it is clear that the C-terminal region exerts negative regulation on Crk signaling and transformation. Whereas models for protein interactions mediated by the SH2 and SH3N have been well documented to explain how Crk functions as an adaptor protein [14,168,174], how the C-terminus of Crk imposes negative regulation has only recently begun to be unraveled [15,175-178]. Earlier biochemical studies suggested that the Crk SH3C could impose regulation on the adaptor protein function of Crk. For example, when expressed in Rat 3Y1 cells, the SH3C domain suppressed p130Cas phosphorylation and the transforming potential of Crk II, and negatively regulated EGF signaling to Ras [179]. Mutations in the SH3C of Crk also lead to increased association of Abl with the SH3N, suggesting that the SH3N and SH3C functionally interact [180]. This latter study also revealed that disruption of the boundary between the SH3 linker and the SH3C domain (aa 240–296 in chicken Crk) regulated the activation of FAK and increased the numbers of focal adhesions in cells. This paradigm for autoinhibition of the SH3 linker and SH3C domain suggested that regions independent of the Y221 might contribute to the negative regulation of Crk.

NMR studies on various Crk proteins that contain the SH3C domain and SH3 linker have begun to offer explanations as to the nature of its regulatory aspects. Like the Y221YAQP motif, the SH3C contributes to the negative regulation of Crk II and CrkL by intramolecular domain crosstalk. Although the Crk SH3C is highly conserved from *C. elegans *to mammals, there are notable differences in the Crk SH3C compared to canonical SH3 domains that bind PxxP motifs. First, although the amino acids that comprise the structural elements of the hydrophobic core are highly conserved, the amino acids that comprise the surface of the SH3C, in particular, where the PxxPxK is expected to bind, are highly divergent [165,176]. The charged residues on the surface of the SH3 domain that interact with the P1, P3, and K6 residues are replaced by unusually polar and bulky amino acids, precluding a socket structure for the prolines and providing a clear explanation for why CrkSH3C does not bind PxxPx(K, R) ligands. Indeed, inspection of the solution NMR structure of the isolated murine SH3C does not show an obvious binding grove, suggesting that this domain may not bind to, or bind with low affinity, to target proteins [176]. Indeed, the only known protein that has been shown to interact with the Crk SH3C domain is the nuclear export protein Crm1, which is posited to bind to a LALEVGELVKV sequence in the Crk SH3C, and has broad similarity to a nuclear export sequence (NES) [181]. However, in the monomer Crk proteins, the LVK motif is buried in the hydrophobic core of the SH3C and not likely to form a contact surface. Interestingly, in the case of the CrkL SH3C, partial SH3 unfolding that may occur during monomer to dimer transition in the nucleus, specifically may expose this LVK sequence for Crm1 binding [175]. This suggests that, in the context of nuclear Crk, regulated unfolding of the SH3C domain may provide a novel and unexpected regulatory mechanism for Crk efflux. Future studies aimed at identification of the molecular mechanisms of Crk unfolding, and whether this reflects an anti-apoptotic mechanism warrant further investigation [182].

**Figure 5:**
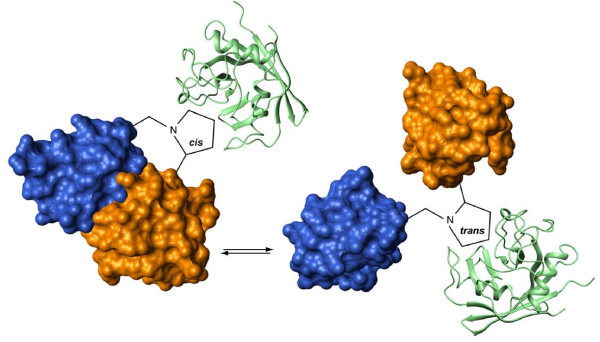
**SH3N and SH3C domain communication in chicken Crk II is mediated by interaction of CyPA**. Prolyl *cis-trans *isomerization centered on P_238_FY regulates intradomain communication between the SH3N and SH3C domains. In the *cis *configuration, the SH3C or Crk forms a closed structure over the SH3N, preventing its association with proline-containing binding partners. In the *trans *configuration, the linker and SH3N are extended, releasing negative regulation. Blue = SH3N; Orange = SH3C; Green = CypA.

**Figure 6:**
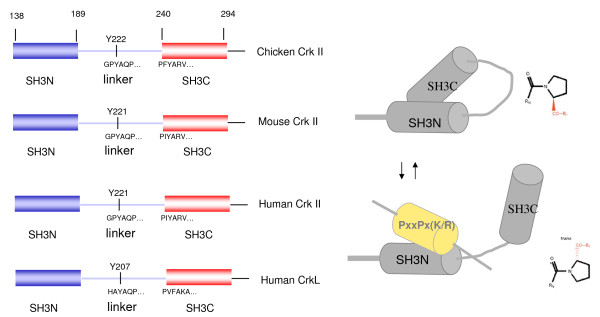
**Sequence alignment of various Crk species**. Chicken Crk is unique in possessing a PFY motif. As alluded to in the text, this may imply that chicken Crk is uniquely regulated by prolyl cis-trans isomerization. Shown in the right side of the figure is a schematic representaion depicting accessibility in the different conformations.

To better understand the mechanism by which the SH3C and linker regulate the SH3N, Kalodimos and coworkers used NMR analysis on a SH3N-linker-SH3C fragment of chicken Crk [178] (Figure 5). In solution at pH 5.5, this fragment showed characteristics of two species in slow exchange originating from *cis-trans *isomerization at the proline from the PFY motif at the boundary of the linker-SH3C, a region previously identified as an important negative regulatory region in chicken Crk [180]. Moreover, the *cis *and *trans *forms appear to have entirely different regulatory functions: the *cis *isomer is locked in a configuration that tethers the SH3N, preventing ligand binding, while the *trans *configuration appears extended and flexible (Figure 5). Support of this model comes from the fact that mutation at either Pro238 or F239 abrogates *cis-trans *isomerization, favoring the *trans *form and leading to increased association of Abl with the SH3N [178]. By sequence analysis, this proline was expected to be a substrate for Cyclophilin A (CypA), a protein that has peptidyl prolyl isomerase activity and catalyzes the prolyl isomerization of peptide bonds. Interestingly, this suggests that enzymatic-catalyzed *cis-trans *proyl isomerization may influence Abl and Arg dependent phosphorylation and hence the rate at which Crk proteins are negatively regulated. Future studies should investigate the physiological significance of CypA on Crk function, as well as whether cyclosporin A (inhibitor of CypA) may be a specific inhibitor of Crk pathways *in vivo*. Another interesting aspect of this regulatory mechanism involving the PFY motif in chicken Crk is that F239, adjacent to P238, was absolutely required to achieve *cis-trans *isomerization, and mutation of F239 to Ile or Val abrogated this regulatory mechanism. This is most intriguing, given the fact that chicken is the only species of Crk with the PFY motif. This motif diverges to PIY in human and PVY in mouse (Figure 6).

**Figure 7:**
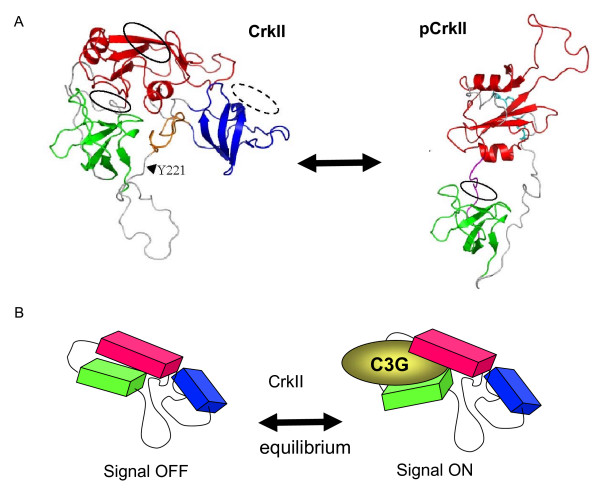
**NMR analysis of the structure of Crk II and pCrkII**. (A) NMR structure of CrkII (1–304) and phosphorylated form of CrkII (1–228). SH2, SH3N, and SH3C are indicated as red, green, and blue colors. Inter SH3 core region (224–237) of CRKII is indicated as yellow. In pCrkII, interface of SH3N is covered with the sequence between SH2 and SH3N (122-133 as magenta). Phosphorylated residue of pYAQP is indicated as light blue. (B). Schematic structure of CrkII with SH3N target C3G. In the cytoplasm, equilibrium of CrkII alone and CrkII/C3G complex may be established.

Recently, Inagaki and colleagues performed NMR of the entire structure of Crk I and Crk II and the phosphorylated form of Crk II without the SH3C (amino acids 1–228) [15] (Figure 7A). Interestingly, while the SH2 and SH3 domains of Crk I are flexible [15], conversely, Crk II forms a compact structure. Solution structure of Crk II showed that the three SH domains were assembled to a central short sequence, residing in the inter SH3 region (amino acids 224–237) called the inter SH3 core region (ISC), and that this compact structure was stabilized mainly by the SH3C domain. These studies also found that the SH3N is semi-closed and covered by the bottom of the SH2 domain, mimicking the interaction of SH3 and PxxPxK. These structures in many respects provide confirmation of previously observed differences in the biological activity of Crk I versus Crk II, including the fact that the affinity of Crk II for the SH3N target polyproline motifs is lower than the affinity of Crk I. Upon phosphorylation, the entire Crk structure is dramatically shifted, and the SH2 binds to phospho-Y221, as reported previously. In phosphorylated Crk II, the SH3N surface was shown to be blocked by an internal sequence between the SH2 and SH3 domains (Arg122- Glu133), suggesting that phosphorylated Crk II cannot bind to either of the SH2 or SH3N targets (Figure 7A). Again, these observations are consistent with previous biochemical and cell biological studies, which predict that: (i) Crk I is constitutively active, (ii) Crk II is regulated by the intramolecular SH3N interaction, and (iii) phosphorylated Crk appears to be completely shut off for binding effector proteins (Figure 7B and additional File 1). Moreover, although details remain to be determined, it appears that the mode of negative regulation for mammalian and chicken Crk II proteins has somewhat diverged.

### Complex Cell Biological Processes regulated by Crk II

#### Role of Crk in Rac1 activation, cell migration, and phagocytosis

A significant advance towards a molecular understanding of Crk biology was revealed following a screen for genes involved in cell-death pathways in *C. elegans *using classical worm genetics [59]. Earlier studies in *C. elegans *elegantly identified genes that mediated the manifestations of apoptosis (Ced-3/Caspases and Ced-4/APAF-1) and in the suppression of apoptosis (Ced-9/Bcl-2) [183,184]. Upon closer examination of the worm phenotypes, a third class of mutations was found, based on their inability to engulf apoptotic corpses, and as such gave a phenotype of persistent corpses. Four of these gene products, representing the dominant pathway for clearance in the worm, were identified as a module consisting of Ced-2, Ced-5, Ced-10, and Ced-12, which have the mammalian counterparts, Crk II, DOCK180, Rac1, and ELMO, respectively [185]. In addition to the defects in cell clearance in the *C. elegans *model, a second related, if not overlapping, phenotype was noted in the Ced-2/Crk deficient worms that involved defects in the migration of cells into the distal tip [59]. Defects in distal tip migration could be rescued by Ced10 (Rac1) or Ced5 (DOCK180), suggesting that Crk was positioned upstream of Rac1 in this pathway. Investigations by Ishimaru and Hanafusa, studying *Drosophila *metamorphosis, elegantly demonstrated that Drosophila Crk (Dcrk) cooperates with DOCK180 (called myoblast city or Mbc) and ELMO for border cell migration in oogenesis and hemocyte migration during embryogenesis, and further mapped this pathway downstream of PVR, the fly equivalent molecule of PDGF Receptor and VEGF-Receptor [157]. In addition to these functions in physiological cell migration, Crk also appears to utilize cell migration and motile responses in pathophysiological functions that include pathogen uptake and virulence, as well as cancer metastasis.

### Crk and bacterial infectious diseases

In recent years, a growing and unexpected body of evidence suggests that Crk may contribute to bacterial pathogenesis by influencing entry into cells or by serving as targets of bacterial toxins that disrupt essential cellular function through Crk-dependent mechanisms. A role for Crk in bacterial uptake pathways was first noted by Weidow and colleagues, who demonstrated that *Yersinia pseudotuberculosis *infection into human epithelial cells activated the p130Cas-Crk-DOCK180-Rac1 pathway via binding of the bacterial invasin to the β1 integrin receptor [63,186]. This pathway appears be somewhat analogous to the phagocytic pathway for apoptotic cells, although the phagocytic pathway involves β3 and β5 integrins. More recent studies by Burton and colleagues found that Crk is also implicated in *Shigella flexneri *infection into NIH 3T3 cells using a curious pathway involving Abl-Arg-mediated Crk Y221 phosphorylation, whereby Crk Y221F mutation blocked *S. Flexneri *infection [64]. In very provocative studies involving *Pseudomonas aeruginosa *infection [187] and *Helicobacter pylori *infection [66], Crk appears to acquire a gain-of-function activity by co-opting activities with bacterial virulence factors. In the former study, a novel role for Abl and Crk phosphorylation was identified and found to be essential for *Pseudomonas aeruginosa *internalization [188]. In the case of *H. pylori*, Crk associates with tyrosine phosphorylated CagA, and the CagA/Crk complex regulates Erk signaling and Rac1-mediated cytoskeletal assemblages [66]. Although in *H. pylori*-associated gastric cancer, CagA-SHP2 signaling is known to play a central role, the functional significance of the CagA/Crk complex for cellular infection should be evaluated. Finally, exoenzyme T (ExoT) of *P. aeruginosa *ADP-ribosylates Crk I and Crk II at specific residues in the SH2 domain (Arg20) that impairs binding of Crk to p130Cas [187]. Although to date, there has been very little to report on the role that Crk proteins play in viral entry and pathogenesis, this may be an area of active ferment as a recent study showed that CrkL can bind to NS1 proteins in Influenza A infected cells [189].

### Crk and human cancers

A number of studies have suggested that Crk may play an important role in human cancers. Immunohistochemical analysis of human cancers showed the over-expression of Crk in adenocarcinomas of lung, breast, and stomach, and also sarcomas, followed by the demonstration of increased levels of Crk mRNA in more aggressive phenotypes of lung adenocarcinoma [62,190-194]. In an analysis of gene-expression profiles of 86 primary lung adenocarcinomas, increased Crk expression was shown to be a predictive factor, contributing to poor prognosis and shorter survival [190]. Overexpression of Crk in tumor cells or in experimental model systems leads to increases in tyrosine phosphorylation of p130Cas and the activation of an intracellular loop that further enhances the activity of Crk and induces increased motility and the aggressive potential of cancer cells. Therefore, Crk proteins are not simply conduits for intracellular signal transduction but also can control the amplitude of signaling.

**Figure 8:**
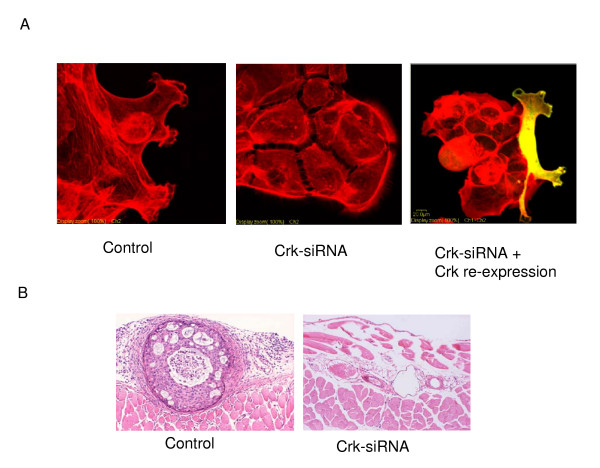
**Effect of Crk siRNA on morphology and biological activity of human ovarian cancer cell line MCAS**. (A). Membrane ruffling is observed in the control MCAS cells (left panel), and siRNA for Crk suppressed these phenotypes (middle). When Crk is overexpressed, clear ruffling appeared (right, yellow color indicates Crk expression). (B). Control MCAS cells form metastatic nodules in the peritoneal cavity mimicking peritonitis carcinomatosa of human ovarian cancer patients when cells are injected into the peritoneal cavity of nude mice. H&E stain for lymphatic invasion of the peritoneal wall is displayed (left). Crk depleted cells by siRNA lose their malignant potential (right). [See additional file 2] (i) Human synovial sarcoma cell line Fuji with control siRNA was stimulated with HGF. (ii) Human synovial sarcoma cell line Fuji with CRK siRNA was stimulated with HGF. Note that the cells exhibit flat morphology and their movement is static.

Based on these observations, several groups have begun to investigate the effects of Crk knockdown on the phenotypic behavior of human tumor cells. Simultaneous downregulation of both Crk I and Crk II by siRNA demonstrated the essential role of Crk in the malignant features of human ovarian cancer cells [192], synovial sarcoma cells [112], and brain tumors, such as glioblastoma cells [58]. Recent studies also suggest that Crk II is a likely target for MiR-126, and that overexpression of MiR-126 in lung cancer cells decreased adhesion, spreading, and invasion [195]. In Crk knockdown cells, formation of focal adhesion was decreased, and cells had cytoskeletal disorganization, whereby formation of laemellipodial structures at the leading edges of cells was impaired (Figure 8, additional file 2 and additional file 3). For example, knockdown of Crk in human synovial sarcoma cells showed a suppression of HGF-dependent activation of Rac1 and decreased motility [192]. In glioblastoma cells, Crk knockdown affected the early attachment to laminin. In addition, Crk also regulated Rho-dependent phosphorylation of ezrin-radixin-moesin proteins that direct migration towards hyaluronic acid in the brain [58]. Increased levels of Crk I mRNA are frequently observed in WHO grade III and IV malignant gliomas [191,193]. Thus, Crk I expression may correlate with poor prognosis, and this is a novel example of splicing-dependent control of human cancer malignancy. As Crk knockdown in cell lines was not lethal, Crk may be an effective therapeutic target for decreasing the metastatic potential of human tumor cells (see additional file 2 and additional file 3).

The above studies suggest that Crk may have a central role in cell motility and metastasis in highly aggressive motile cancer cells. There are a plethora of studies showing that p130Cas-HEF and the FAK-Src circuitry is involved in tumorigenesis, and Crk may indeed be an attractive target due to its central integrative downstream role in signaling by these molecules. Recent studies now suggest that DOCK180 and/or ELMO may be oncogenes, or have oncogenic potential, and Crk may be an attractive drug target in tumor cells that use Rac1 activation to acquire motile or metastatic potential. DOCK180, for example, is overexpressed at the borders, but not at the centers, of human malignant glioma, and together with ELMO1, it regulates Rac activity to enhance tumor invasion [196]. Although the relation of C3G and human cancer is controversial, an increase in C3G expression is observed in human small cell carcinoma [197], and a Crk-C3G-Rap1 pathway leading to B-RAF and Erk activation is involved in transformation downstream of Ret in papillary thyroid carcinoma [124]. In addition, C3G activation of Rap1 regulates a number of cell-matrix or cell-cell interactions through integrins and cadherins [130], pointing to a role for C3G overexpression in the modulation of adherens junctions, leading to tumor cell dissemination [130,198]. It will be important to experimentally observe whether siRNA toward Crk can influence the motile behavior of cells that express upregulated C3G and/or Rap-1. Finally, a most curious function for CrkL has been recently proposed, whereby a secreted form of CrkL was shown to bind to the plexin-semaphorin-integrin (PSI) domain of β1 integrin at the extracellular domain in order to promote cell growth and survival [213]. These authors made the provocative suggestion that targeting extracellular Crk proteins may have therapeutic value.

Another area of clinical investigation is the role of tyrosine phosphorylation of CrkL and Crk II in CML [199-201]. This cancer results from the chromosomal translocation termed Philadelphia chromosome (Ph1) as t(9;22), generating the aberrant chimeric protein Bcr-Abl. Current understanding is that CrkL is not essential for the development of CML, because CrkL knockout mice developed CML by the introduction of Bcr-Abl. However, the full role of CrkL and Crk II in CML has yet to be explained.

### Crk and pro-apoptotic pathways

In recent years, both Crk II and CrkL have been implicated in nuclear signal transduction events. For CrkL, elegant studies have shown that CrkL binds tyrosine phosphorylated Stat5 in Bcr-Abl expressing cells, and in a variety of cytokine-stimulated hematopoietic cells [202,203]. In many, if not all, of these cases, the complex translocates to the nucleus to bind STAT5-responsive elements [204,205]. Although Crk II can also bind to STAT5, this complex does not appear to enter the nucleus, nor do anti-Crk II antisera supershift STAT5 DNA binding complexes [34]. While these studies may indicate that CrkL has a role in transcription of interferon regulated genes, interesting studies by Kornbluth and colleagues indicate that Crk may actively and directly participate in apoptosis in *Xenopus *by activating caspases [181]. Depletion of Crk II from egg extracts prevented apoptosis, and these studies further demonstrated that Crk binds to the cell cycle regulatory protein Wee1, implying that this pro-apoptotic function of Crk II may be due to nuclear localization [206]. Evidence in support of this model includes the fact that Crk has a putative NES in the SH3C domain, that when mutated increases cell death, and that ionizing radiation increases complex formation between Wee1 and Crk II. Furthermore, evidence for a direct role of Crk in apoptosis is supported by the findings that specific targeting of Crk to the nucleus by fusing NLS sequences, spontaneously activates apoptosis and potentiates stimuli that induce apoptosis [182]. Recent structural studies with CrkL indicate that the Crk NES may only function in the dimeric state of the protein, mediated by a homotypic interaction in the SH3C domains [175]. These studies predict that the Crk NES motif may become exposed conditionally under certain circumstances, an event that would actively promote efflux of a nuclear Crk pool into the cytoplasm. However, the presence of an NES sequence in Crk II does not explain how Crk II initially trafficks to the nucleus, since Crk does not appear to possess any NLS sequences. It will be equally important to characterize the physiological signals that result in Crk translocation into the nucleus, and whether there exists a regulatory signal that controls the interaction between Crk and Crm1.

### Future directions and new frontiers

The Crk gene was identified about 20 years ago, and the characterization of Crk and its mode of cell signaling has arguably revolutionized the way we think about intracellular signal transduction. Despite significant advances and an enormous wealth of new information, the field is now faced with the challenging problem of how these complex signaling networks are controlled in time and space to produce biological responses. Efforts will need to be redirected from the relatively simple task of identifying specific effector proteins to an understanding of when and where these complexes are formed in cells. Development of FRET technology that reports specific protein-protein interactions, as well as the generation of a battery of phosphospecific antiseras against specific post-translational epitopes, should help provide a better understanding of Crk biology. This exciting field can expect substantial progress as the new observations continue. One thing is certain: the field of Crk biology will continue to grow over the next 20 years.

## Competing interests

The authors declare that they have no competing interests.

## Authors' contributions

RB wrote the background and significance and the sections on the biological significance of Crk. CK and FI wrote parts of the sections on Crk structure. ST wrote the sections on Crk and Cancers. All authors read and approved the final manuscript.

## Supplementary Material

Additional file 1 **A rotational view of the Crk II structure is shown in the supplemental data**. The organization of the SH2 and SH3 domains are indicated in order to illustrate negative regulation. Click here for file

Additional file 2 **Phenotype of Crki expressing cells**. When Crk is downmodulated by siRNA, cells exhibit decreased motility and invasion. Click here for file

Additional file 3 **Phenotype of Crki expressing cells**. When Crk is downmodulated by siRNA, cells exhibit decreased motility and invasion. Click here for file
