# Supplementary figures and images for: Crk and CrkL adaptor proteins: networks for physiological and pathological signaling
Source: Cell Commun Signal. 2009 May 10;7:13. doi: 10.1186/1478-811X-7-13 (PMC2689226; doi:10.1186/1478-811X-7-13)

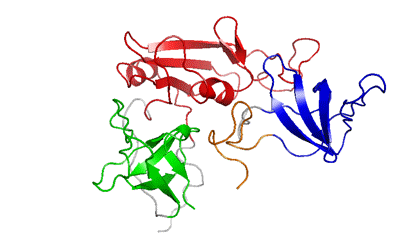

Supplement: Additional file 1 — A rotational view of the Crk II structure is shown in the supplemental data. The organization of the SH2 and SH3 domains are indicated in order to illustrate negative regulation. [file 1478-811X-7-13-S1.gif]
